# Supplementary material for: Influence of selected non-antibiotic pharmaceuticals on antibiotic resistance gene transfer in Escherichia coli
Source: PLoS One. 2024 Jun 21;19(6):e0304980. doi: 10.1371/journal.pone.0304980 (PMC11192386; doi:10.1371/journal.pone.0304980)
Supplement: S1 File — (DOCX) [file pone.0304980.s001.docx]

**Supplementary file**

S**1: PCR conditions and primer sequences**

|  | Primer sequences | PCR conditions | Ref. |
| --- | --- | --- | --- |
| *traF* | Forward:  AAGTGTTCAGGGTGCTTCTGC  Reverse:  GTCGCCTTAACCGTGGTGTT | Initial denaturation (94ºC for 4 min.), 35 cycles (94ºC for 30 sec., 55ºC for 30 sec., 72ºC for 1 min), 7 min final extension | **^1^** |
| *tetA* | Forward: GACTATCGTCGCCGCACTTA  Reverse:  ATAATGGCCTGCTTCTCGCC | Initial denaturation (94ºC for 4 min.), 30 cycles (94ºC for 30 sec., 54ºC for 30 sec., 72ºC for 1 min), 7 min final extension | **^1^** |
| *strA*-*strB* | Forward  TATCTGCGATTGGACCCTCTG  Reverse  CATTGCTCATCATTTGATCGGCT | Initial denaturation (95ºC for 60 s), 30 cycles (95ºC for 60 s, 60ºC for 30 s ,72ºC for 60 s). | **^2^** |

1. Wang Y, Lu J, Zhang S, et al. Non-antibiotic pharmaceuticals promote the transmission of multidrug resistance plasmids through intra-and intergenera conjugation. *The ISME journal.* 2021;15(9):2493-2508.

2. Sunde M, Norström M. The genetic background for streptomycin resistance in Escherichia coli influences the distribution of MICs. *Journal of Antimicrobial Chemotherapy.* 2005;56(1):87-90.
