# Supplementary material for: Influence of selected non-antibiotic pharmaceuticals on antibiotic resistance gene transfer in Escherichia coli
Source: PLoS One. 2024 Jun 21;19(6):e0304980. doi: 10.1371/journal.pone.0304980 (PMC11192386; doi:10.1371/journal.pone.0304980)
Supplement: Attachment — Submitted filename: PONE-May-25-2023-Revsisions.pdf [file pone.0304980.s003.pdf]

PLOS ONE

Role of Some Antiemetics and Antispasmodics in the dissemination of antibiotic resistance genes among intestinal pathogenic E. coli

Please write it as the complete name, Escherichia coli

--Manuscript Draft--

**Manuscript Number:**	PONE-D-23-09629
**Article Type:**	Research Article
**Full Title:**	Role of Some Antiemetics and Antispasmodics in the dissemination of antibiotic resistance genes among intestinal pathogenic E. coli
**Short Title:**	Antiemetics and antispasmodics role in the dissemination of antibiotic resistance among intestinal pathogens
**Corresponding Author:**	Rehab Mahmoud Abd El-Baky, Professor<br>Minia University<br>Minia, EGYPT
**Keywords:**	Antibiotic resistance genes; non-antibiotics; anti-emetics; anti-spasmodic; conjugation; metoclopramide HCl; hyoscine butyl bromide; tiemonium methylsulfate.
**Abstract:**	Background: Antibiotic resistance genes (ARGs) transfer rapidly among bacterial species all over the world contributing to the aggravation of antibiotic resistance crisis. Antibiotics at sub-inhibitory concentration induce horizontal gene transfer (HRT) between bacteria, especially through conjugation. The role of common non-antibiotic pharmaceuticals in the market in disseminating antibiotic resistance is not well studied.<br>Objectives: In this work, we indicated the effect of some commonly used non-antibiotics including antiemetic (metoclopramide HCl) and antispasmodics (hyoscine butyl bromide and tiemonium methyl sulfate) on the plasmid-mediated conjugal transfer of antibiotic resistance genes between pathogenic E. coli in the GIT.<br>Methods: Broth microdilution assay was used to test the antibacterial activity of the tested non-antibiotics. A conjugation mating system was applied in presence of the studied non-antibiotics to test their effect on conjugal transfer frequency. Plasmid extraction and PCR were performed to confirm the conjugation process. Transmission electron microscopy (TEM) was used for imaging the effect of non-antibiotics on bacterial cells.<br>Results: No antibacterial activity was reported for the used non-antibiotics. Plasmid-mediated conjugal transfer between isolates was induced by metoclopramide HCl but suppressed by hyoscine butyl bromide. Tiemonium methylsulfate slightly promoted conjugal transfer. Aggregation between cells and periplasmic bridges was clear in the case of metoclopramide HCl while in presence of hyoscine butyl bromide little affinity was observed.<br>Conclusion: This study indicates the contribution of non-antibiotic pharmaceuticals to the dissemination and evolution of antibiotic resistance in the environment at the community level. Metoclopramide HCl showed an important role in the spread of antibiotic resistance.
**Order of Authors:**	Doaa S Mohamed
	Rehab Mahmoud Abd El-Baky, Professor
	Mohamed Elmokhtar
	Sahar K Ghanem
	Ramadan Y Hamed
	Alaa M. Alqahtani
	Mohammed A. S. Abourehab
	Eman F Ahmed
**Additional Information:**	
**Question**	**Response**
**Financial Disclosure**	The funders had no role in study design, data collection and analysis, decision to

Enter a financial disclosure statement that describes the sources of funding for the work included in this submission. Review the submission guidelines for detailed requirements. View published research articles from *PLOS ONE* for specific examples.<br><br>This statement is required for submission and **will appear in the published article** if the submission is accepted. Please make sure it is accurate.<br><br>**Unfunded studies**<br>Enter: *The author(s) received no specific funding for this work.*<br><br>**Funded studies**<br>Enter a statement with the following details:<br>• Initials of the authors who received each award<br>• Grant numbers awarded to each author<br>• The full name of each funder<br>• URL of each funder website<br>• Did the sponsors or funders play any role in the study design, data collection and analysis, decision to publish, or preparation of the manuscript?<br>• **NO** - Include this sentence at the end of your statement: *The funders had no role in study design, data collection and analysis, decision to publish, or preparation of the manuscript.*<br>• **YES** - Specify the role(s) played.<br><br>* typeset	publish, or preparation of the manuscript
**Competing Interests**<br><br>Use the instructions below to enter a competing interest statement for this submission. On behalf of all authors, disclose any competing interests that could be perceived to bias this work—acknowledging all financial support and any other relevant financial or non-financial competing interests.<br><br>This statement is required for submission and **will appear in the published article** if	The authors have declared that no competing interests exist.

the submission is accepted. Please make sure it is accurate and that any funding sources listed in your Funding Information later in the submission form are also declared in your Financial Disclosure statement.<br><br>View published research articles from *PLOS ONE* for specific examples.<br><br>**NO authors have competing interests**<br><br>Enter: *The authors have declared that no competing interests exist.*<br><br>**Authors with competing interests**<br><br>Enter competing interest details beginning with this statement:<br><br>*I have read the journal's policy and the authors of this manuscript have the following competing interests: [insert competing interests here]*<br><br>* typeset	
**Ethics Statement**<br><br>Enter an ethics statement for this submission. This statement is required if the study involved:<br><br>• Human participants<br>• Human specimens or tissue<br>• Vertebrate animals or cephalopods<br>• Vertebrate embryos or tissues<br>• Field research<br><br>Write "N/A" if the submission does not require an ethics statement.<br><br>General guidance is provided below. Consult the submission guidelines for detailed instructions. **Make sure that all information entered here is included in the Methods section of the manuscript.**	The study was conducted according to the guidelines of the Declaration of Helsinki, priori approval (No. 2/2022) by the ethical committee of Faculty of Pharmacy, Deraya University. Informed consent was not required since bacterial isolates were the subject of this study. Isolates, not human beings were studied. Thus, informed consent was not required by the local Ethics Committee

Powered by Editorial Manager® and ProduXion Manager® from Aries Systems Corporation

**Format for specific study types**<br><br>**Human Subject Research (involving human participants and/or tissue)**<br>• Give the name of the institutional review board or ethics committee that approved the study<br>• Include the approval number and/or a statement indicating approval of this research<br>• Indicate the form of consent obtained (written/oral) or the reason that consent was not obtained (e.g. the data were analyzed anonymously)<br><br>**Animal Research (involving vertebrate animals, embryos or tissues)**<br>• Provide the name of the Institutional Animal Care and Use Committee (IACUC) or other relevant ethics board that reviewed the study protocol, and indicate whether they approved this research or granted a formal waiver of ethical approval<br>• Include an approval number if one was obtained<br>• If the study involved *non-human primates*, add *additional details* about animal welfare and steps taken to ameliorate suffering<br>• If anesthesia, euthanasia, or any kind of animal sacrifice is part of the study, include briefly which substances and/or methods were applied<br><br>**Field Research**<br><br>Include the following details if this study involves the collection of plant, animal, or other materials from a natural setting:<br>• Field permit number<br>• Name of the institution or relevant body that granted permission	
**Data Availability**<br><br>Authors are required to make all data underlying the findings described fully available, without restriction, and from the time of publication. PLOS allows rare exceptions to address legal and ethical concerns. See the PLOS Data Policy and FAQ for detailed information.	Yes - all data are fully available without restriction

A Data Availability Statement describing where the data can be found is required at submission. Your answers to this question constitute the Data Availability Statement and **will be published in the article**, if accepted.<br><br>**Important:** Stating 'data available on request from the author' is not sufficient. If your data are only available upon request, select 'No' for the first question and explain your exceptional situation in the text box.<br><br>Do the authors confirm that all data underlying the findings described in their manuscript are fully available without restriction?	
**Describe where the data may be found in full sentences. If you are copying our sample text, replace any instances of XXX with the appropriate details.**<br><br>• If the data are **held or will be held in a public repository**, include URLs, accession numbers or DOIs. If this information will only be available after acceptance, indicate this by ticking the box below. For example: *All XXX files are available from the XXX database (accession number(s) XXX, XXX.).*<br>• If the data are all contained **within the manuscript and/or Supporting Information files**, enter the following: *All relevant data are within the manuscript and its Supporting Information files.*<br>• If neither of these applies but you are able to provide **details of access elsewhere**, with or without limitations, please do so. For example:<br><br>*Data cannot be shared publicly because of [XXX]. Data are available from the XXX Institutional Data Access / Ethics Committee (contact via XXX) for researchers who meet the criteria for access to confidential data.*<br><br>*The data underlying the results presented in the study are available from (include the name of the third party*	All relevant data are within the manuscript and its Supporting Information files.

and contact information or URL).<br>• This text is appropriate if the data are owned by a third party and authors do not have permission to share the data.<br><br>* typeset	
Additional data availability information:	

Manuscript Click here to access/download;Manuscript;Manuscript.docx

Title Page

Role of Some Antiemetics and Antispasmodics in the dissemination of antibiotic resistance genes among intestinal pathogenic *E. coli*

Write the genus of bacteria as complete nomenclature, Escherichia coli

Doaa Safwat Mohamed[1], Rehab Mahmoud Abd El-Baky[2,3*], Mohamed Ahmed El-Mokhtar[4], Sahar K Ghanem[5], Ramadan Yahia[3], Alaa M. Alqahtani[6], Mohammed A. S. Abourehab[7] and Eman Farouk Ahmed[1]

[1]Microbiology & Immunology Department, Faculty of Pharmacy, Sohag University, Egypt
dd_25_sfwt@yahoo.com & eman.farouk@pharm.sohag.edu.eg

[2]Microbiology & Immunology Department, Faculty of Pharmacy, Minia University, Egypt

[3]Microbiology and Immunology Department, Faculty of Pharmacy, Deraya University, Minia, Egypt. ramadan.yahia@deraya.edu.eg

[4]Medical Microbiology & Immunology Department, Faculty of Medicine, Assiut University, Egypt. Elmokhtarma@aun.edu.eg

[5]Pharmacology & Toxicology Department, Faculty of Pharmacy, Sohag University, Egypt. saharkhm2002@yahoo.com

[6]Pharmaceutical Chemistry Department, College of Pharmacy, Umm Al-Qura University, Makkah 21955, Saudi Arabia; amqahtani@uqu.edu.sa

[7]Pharmaceutics Department, College of Pharmacy, Umm Al-Qura University, Makkah 21955, Saudi Arabia; maabourehab@uqu.edu.sa.

*Coresponding author: Rehab Mahmoud Abd El-Baky

Professor of Microbiology and Immunology, faculty of pharmacy, Deraya university

Professor of Microbiology and Immunology, faculty of pharmacy, Minia University

E-mail: rehab.mahmoud@mu.edu.eg

Mobile:02-01092487412

Role of Some Antiemetics and Antispasmodics in the dissemination of antibiotic resistance genes among intestinal pathogenic *E. coli*

Abstract

Background: Antibiotic resistance genes (ARGs) transfer rapidly among bacterial species all over the world contributing to the aggravation of antibiotic resistance crisis. Antibiotics at sub-inhibitory concentration induce horizontal gene transfer (HRT) between bacteria, especially through conjugation. The role of common non-antibiotic pharmaceuticals in the market in disseminating antibiotic resistance is not well studied.

Objectives: In this work, we indicated the effect of some commonly used non-antibiotics including antiemetic (metoclopramide HCl) and antispasmodics (hyoscine butyl bromide and tiemonium methyl sulfate) on the plasmid-mediated conjugal transfer of antibiotic resistance genes between pathogenic *E. coli* in the GIT.

Methods: Broth microdilution assay was used to test the antibacterial activity of the tested non-antibiotics. A conjugation mating system was applied in presence of the studied non-antibiotics to test their effect on conjugal transfer frequency. Plasmid extraction and PCR were performed to confirm the conjugation process. Transmission electron microscopy (TEM) was used for imaging the effect of non-antibiotics on bacterial cells.

Results: No antibacterial activity was reported for the used non-antibiotics. Plasmid-mediated conjugal transfer between isolates was induced by metoclopramide HCl but suppressed by hyoscine butyl bromide. Tiemonium methylsulfate slightly promoted conjugal transfer. Aggregation between cells and periplasmic bridges was clear in the case of metoclopramide HCl while in presence of hyoscine butyl bromide little affinity was observed.

Conclusion: This study indicates the contribution of non-antibiotic pharmaceuticals to the dissemination and evolution of antibiotic resistance in the environment at the community level. Metoclopramide HCl showed an important role in the spread of antibiotic resistance.

Delete "in the environment"

Keywords: Antibiotic resistance genes, non-antibiotics, anti-emetics, anti-spasmodic, conjugation, metoclopramide HCl, hyoscine butyl bromide, tiemonium methylsulfate.

Please, delete some key words to decrease the number of key words

1. Introduction

One of the most challenges or the biggest threats as described by the World health organization (WHO) is the emergence of antimicrobial resistance (AMR). As WHO said

that if no action is taken against the high increase in antibiotic resistance, more than 10 million people will die by 2050 [1]. Extensive use of antibiotics causes selective pressure on microbes giving rise to the emergence of resistant cells [2-7]. Horizontal gene transfer (HGT) is one of the major means of transferring or disseminating antibiotic resistance genes (ARGs) in the surrounding environment. Horizontal gene transfer can occur by conjugation, transduction, and transformation. Regarding conjugation, the exchange of genetic material between the donor and the recipient occurs by direct cell to- cell contact or via a connecting pilus [8, 9]. Typically, the exchange is mediated by mobile genetic elements, such as a conjugative plasmid. In addition, it was found that it is the most common way for disseminating antimicrobial resistance. Despite the spontaneous frequency of conjugation being rare, extensive use of antibiotics especially at concentrations lower than their MICs may lead to an increase in the frequency of the conjugation process [10, 11]. Accordingly, looking for unique conjugation inhibitors is an essential challenge in the fight against the spread of antibiotic resistance genes for improving the bacterial response toward antibiotics [12, 13]. Surprisingly, it was recently documented that the consumption of non-antibiotic prescribed drugs represents about ninety fifth of the drug market [14], the role of these prescribed drugs in the evolution of antibiotic resistance has received comparatively very little attention [15]. It remains unknown whether non-antibiotic prescribed drugs promote conjugation between bacterial strains or not which will be of clinical concern, as conjugative multidrug resistance plasmids enable quick expression of multidrug resistance phenotypes, therefore facilitating the emergence and widespread of antibiotic-resistant microorganisms [16]. Additionally, it's not clear whether there are common properties between the non-antibiotic prescribed drugs, or shared mechanisms that promote the ARGs horizontal transfer [11].

Gastro-intestinal tract (GIT) infection is one of the most common infections worldwide. *Escherichia coli*, a member of the microorganism family of Enterobacteriaceae, is the most prevailing commensal dweller of the gastrointestinal tracts of humans and warm-blooded animals, as well as one of the most important pathogens inflicting GIT infection [17]. Diarrheagenic pathotypes of *E. coli* (enterotoxigenic *E. coli* [ETEC], enteropathogenic *E. coli* [EPEC], enteroinvasive *E.*

Add with the reference 17 the following update reference after addition to GIT problem caused by H pylori and Clarithromycin resistance:-

Hussein RA, Al-Ouqaili MTS, Majeed YH. (2021). Detection of Helicobacter Pylori infection by invasive and non-invasive techniques in patients with gastrointestinal diseases from Iraq: A validation study. PLoS One. 23;16(8):e0256393. doi: 10.1371/journal.pone.0256393. PMID: 34424925; PMCID: PMC8382163.

coli [EIEC], and enteroaggregative *E. coli* [EAEC]) are highly detected [18]. Patients suffering from these infections receive antibiotics beside GIT concomitant pharmaceuticals such as, antiemetic and antispasmodic drugs. These drugs are metoclopramide hydrochloride, hyoscine butyl bromide and tiemonium methyl sulfate. Metoclopramide is usually directed to treat a large variety of epithelial duct disorders [19]. It may be used for the treatment of nausea and physiological reaction [20]. Hyoscine butyl bromide is usually used as associate in spasmolytic treatment for the pain and discomfort iatrogenic by abdominal cramps [21].

Tiemonium methyl sulfate is an antimuscarinic quaternary ammonium agent with peripheral atropine similar effect and is employed within the relief of visceral spasms [22]. The transfer of conjugative plasmids may be affected by these prescribed pharmaceuticals. However, the effect of these non-antibiotic pharmaceuticals on bacterial resistance or antibiotic activity remains unknown. In this work, we study the effect of metoclopramide HCl as a commonly used antiemetic and hyoscine butyl bromide and tiemonium methylsulfate as commonly consumed antispasmodics on conjugal transfer frequency among pathogenic *E. coli* isolates in the GIT.

2. Materials and Methods

2.1 Bacterial isolates and reagents:

Four *E. coli* strains were used for conjugative transfer testing. Two strains were obtained from Hiper® bacterial conjugation kit (HTM004, HiMedia). Donor *E. coli* strain (Product code: TKC181) and Recipient *E. coli* strain (Product Code: TKC182). Two *E. coli* strains were isolated from patients suffering from gastrointestinal tract infection, *E. coli*-1 with plasmid harboring resistance gene for tetracycline was the donor and *E. coli*-5 with high chromosomally encoded resistance to streptomycin was used as the recipient.

Raw materials of streptomycin sulphate (Product code: RM220) and tetracycline hydrochloride (Product code: RM219) were included in Hiper® bacterial conjugation kit (HTM004, HiMedia). Hyoscine butyl bromide, metoclopramide HCl were obtained from Sigma Aldrich (USA) while tiemonium methyl sulfate was purchased from Medica Pharma Specialty Pharmaceutical Chemicals (Netherlands).

2.2 Preparation of isolates for the study:

Strains obtained from Hiper® bacterial conjugation kit, Donor strain A was streaked on 2 LB plates with tetracycline (30µg/mL) and the Recipient strain was streaked on LB plates with streptomycin (100µg/mL) and incubated at 37□C overnight.

For clinical strains, Isolation of *E. coli* strains was performed by MacConkey's agar and confirmed biochemically with various screening tests. Disc method was done to select an *E. coli* isolate (Resistant only to tetracycline) and another one (Resistant only to streptomycin) to be used as donor and recipient respectively. Both genomic and plasmid DNA were extracted from the selected isolates (Qiagen plasmid plus midi kit and Qiaprep spin miniprep kit, USA) and polymerase chain reaction was used to amplify tetracycline and streptomycin resistance genes and *traF* gene (encoded by conjugative plasmid and required for pilus assembly and plasmid transfer). PCR primer sequences and conditions are listed in table (1).

Table (1): PCR conditions and primer sequences

	Primer sequences	PCR conditions	Ref.
traF	Forward:<br>AAGTGTTCAGGGTGCTTCTGC<br>Reverse:<br>GTCGCCTTAACCGTGGTGTT	Initial denaturation (94ºC for 4 min.), 35 cycles (94ºC for 30 sec., 55ºC for 30 sec., 72ºC for 1 min), 7 min final extension	**[11]**
tetA	Forward: GACTATCGTCGCCGCACTTA<br>Reverse:<br>ATAATGGCCTGCTTCTCGCC	Initial denaturation (94ºC for 4 min.), 30 cycles (94ºC for 30 sec., 54ºC for 30 sec., 72ºC for 1 min), 7 min final extension	**[11]**

strA-strB	Forward<br>TATCTGCGATTGGACCCTCTG<br>Reverse<br>CATTGCTCATCATTTGATCGGCT	Initial denaturation (95ºC for 60 s), 30 cycles(95ºC for 60 s, 60ºC for 30 s ,72ºC for 60 s).	**[23]**

2.3 Testing antibacterial activity and MIC detection:

Minimum inhibitory concentrations (MICs) of tetracycline, Streptomycin and non-antibiotic pharmaceuticals were tested using broth microdilution assay. The 96-well plates were incubated at 37 °C for 20 h before the OD600 was measured on the plate reader. Wells containing sterile phosphates buffer saline were used as blank. MICs of the bacterial strains were determined as the concentration of the tested agent which inhibited 90% of the growth. Each bacterial strain under the inhibition of the different agents was tested at least in triplicate Hyoscine butylbromide, metoclopramide HCl and tiemonium methyl sulfate were chosen as the non-antibiotic pharmaceuticals for this study. Concentrations of antibiotics were serially diluted from 8 to 0.03125 μg/ml. Also, non-antibiotic pharmaceuticals were used in therapeutically allowed concentrations [24].

2.4 Conjugative transfer in absence and in presence of non-antibiotics:

2.4.1 Conjugative transfer in absence of non-antibiotics:

A single colony from both donor and recipient strains was inoculated in 6ml of LB broth. Then, incubated at 37□C overnight. One ml of overnight grown culture of standard strains or clinical strains was added to 25 ml LB broth with tetracycline. Three ml of overnight culture of the tested strains were added to 25 ml of LB with streptomycin. Cultures were overnight incubated at 37□C in a shaker. Two hundred μL of each donor and recipient cultures were added to a sterile test tube labeled as conjugated sample after gentle mixing and incubated for 1.5 hr without shaking. Two hundred μL of respective cultures were added to tubes labeled as donor and recipient with gentle mixing and incubated for 1.5hr without shaking. Then, 0.1 ml of each culture was plated on

antibiotic containing culture media (one plate containing tetracycline, another plate containing streptomycin and the third containing both tetracycline and streptomycin to isolate transconjugants). Transconjugant colonies' number was divided by donor colonies' number to calculate the conjugative transfer frequency. The value of frequency of conjugal transfer was the average of four different trials. Growth of donor, recipient and transconjugants was not included in calculation due to absence of provided nutrients during the conjugation process [25].

2.4.2 Conjugative transfer in the presence of non-antibiotics:

This work designed a mating model to detect the effect of non-antibiotic pharmaceuticals on conjugative transfer between the tested *E. coli* strains. This was achieved by growing donor and recipient isolates to an OD of 1.8 at 600 nm and mixing donor and recipient in nutrient broth (1:2) ratio in the presence of the following sub-inhibitory concentrations: 0.265 µg/ml (Equivalent to plasma concentration) , 0.53 µg/ml, 1.06 µg/ml, 2.12 µg/ml and 4.24 µg/ml for metoclopramide HCl. For hyoscine butyl bromide; 0.005 µg/ml (Equivalent to plasma concentration), 0.01 µg/ml, 0.02 µg/ml, 0.04 µg/ml and 0.08 µg/ml were used. Finally, tiemonium methyl sulfate has sub-inhibitory concentrations of 0.8 µg/ml (plasma concentration), 1.6 µg/ml, 3.2 µg/ml, 6.4 µg/ml and 12.8 µg/ml. Levofloxacin was used at its sub-MIC as a positive control for conjugative transfer (0.125 µg/ml) [26]. After incubation for 3 hr. at 37ºC, mixture of donor and recipient (20 µL) was plated on tetracycline-streptomycin nutrient agar plates to count colonies of transconjugants. Transconjugant colonies' number was divided by donor colonies' number to calculate the conjugative transfer frequency. The value of frequency of conjugal transfer was the average of four different trials **[11]**.

2.5 Reverse conjugation experiment in the presence of non-antibiotic agents:

Transconjugant bacteria were considered as donor cells. Standard and clinical recipients described previously were used for the conjugate mating system. All strains were tested using different concentrations of the tested agents. Then, mixed by vertexing and incubated for 3h at 37□C without shaking. New transconjugants were used to inoculate agar plates containing antibiotics. Colonies were counted and the frequency of transfer was determined as prescribed above [11].

2.6 Verification of conjugative plasmid transfer:

- **2.6.1 Determination of Minimum inhibitory concentration of transconjugants:**

Minimum inhibitory concentrations (MICs) of transconjugants against tetracycline and streptomycin were determined by broth microdilution methods. The resulting MICs of transconjugants were compared to donor and recipient cells' MICs [24]**.**

- **2.6.2 Detection of plasmids:**

Colonies of transconjugants on selective tetracycline-streptomycin agar plates were picked and stored at -80ºC in glycerol stock (30%). Qiagen plasmid plus midi kit (Qiagen, USA) was applied for extraction of plasmids from donor and transconjugants. PCR was used to amplify plasmid-encoded *traF* and *tetA* genes in both donor and transconjugant. Agarose gel electrophoresis (1%) was used to observe plasmids and amplicons [25].

- **2.7 Transmission Electron Microscopy (TEM):**

Effect of non-antibiotic pharmaceuticals on conjugative activity was studied using TEM (JEM1010, JEOL, Tokyo, Japan). After performing conjugation experiment with non-antibiotic pharmaceuticals and incubation for 24 hr, samples for TEM were collected and imaged. Also, a control sample in absence of non-antibiotics was included. Samples were prepared for TEM according to standard guidelines **[27]**.

2.8 Statistical analysis

Statistical analysis was performed using GraphPad version 8.3.0 software. All data were obtained from at least three biological replicates and presented as mean ± SD. Unpaired *t*-test (normally distributed data) between two groups or one-way ANOVA among multiple groups were used to calculate *P*-values. Differences with $P < 0.05$ were considered significant [28].

3. Results

3.1 Antibacterial activity of non-antibiotic pharmaceuticals:

Broth microdilution assay illustrated no inhibitory activity for metoclopramide, hyoscine butyl bromide and tiemonium methyl sulfate (MIC > 1024 μg/ml). Agar well diffusion assay was used to confirm the previous findings by reporting no inhibitory activity for the non-antibiotic pharmaceuticals against donor and recipient isolates (Figure 1).

Figure 1: Antibacterial activity of non-antibiotic pharmaceuticals: A) against donor isolate, B) against recipient isolate.

3.2 Non-antibiotic pharmaceuticals have different effects against ARGs conjugative transfer:

For both standard and clinical *E. coli* strains; it was found that for the addition of non-antibiotic pharmaceuticals, metoclopramide HCl, at all five concentrations (from 0.265 to 4.24 μg/ml), the number of transconjugants dramatically increased ($P < 0.05$). Hyoscine butyl bromide, at concentrations from 0.005 to 0.01 μg/ml, slightly increased transconjugant number ($P < 0.05$). In contrast, tiemonium methyl sulfate at concentrations from 0.8 to 12.8 μg/ml, decreased the transconjugant number ($P < 0.05$).

Fig. 2a illustrates transconjugant colonies in absence of antibiotics and non-antibiotics as a control. Efficiency of conjugation between donor and recipient isolates was greatly promoted by metoclopramide HCl (Fig. 2b) while was dramatically decreased by hyoscine butylbromide (Fig. 2c). Also, slight induction of conjugal transfer by tiemonium methylsulfate was observed (Fig. 2d). Levofloxacin at sub-MIC level greatly increased conjugal transfer compared to control conjugation tube (Fig 2e). Transfer ratio (expressed as number of transconjugants per recipient cells) and fold change of transfer ratio were illustrated for the tested non-antibiotic agents at different concentrations and levofloxacin at sub-MIC (Table 2). Fold change of transfer ratio was greatly enhanced in presence of metoclopramide HCl (Figure 3) and slightly increased with tiemonium methylsulfate (figure 4). However, it was decreased in presence of hyoscine butylbromide as compared to negative control (Figure 5).

Figure (2): Effect of antibiotics and non-antibiotic pharmaceuticals on conjugal transfer frequency: A) Control, B) Metoclopramide HCl, C) Hyoscine butylbromide D) Tiemonium methylsulfate, E) Levofloxacin at sub-MIC

Table (2): Conjugative transfer under the exposure of different concentrations of non-antibiotic pharmaceuticals

	Number of recipients in 1 ml	Absolute number of transconjugants in 1 ml	Fold change of transconjugant absolute number	Transfer ratio (number of transconjugant / number of recipient)	Fold change of Transfer ratio
**Control**	2.5 x 10^4±817	74±5.72	1.00±0.08	29.7 x 10^{-4}±3 x 10^{-4}	1.00±0.11
**Metoclopramide HCl**					
0.265 µg/ml	2.3 x 10^4±12415	485±8.16	6.55±0.4	195 x 10^{-4}±16 x 10^{-4}	6.55±0.16*
0.53 µg/ml	2.4 x 10^4±12034	523±16.33	7.06±0.33	238 x 10^{-4}±31 x 10^{-4}	8.2±1.5*
1.06 µg/ml	2.2 x 10^4±11002	578±81.65	7.81±0.51	240 x 10^{-4}±23 x 10^{-4}	7.97±0.2*
2.12 µg/ml	2.5 x 10^4±12472	634±27.76	8.57±1.05	255 x 10^{-4}±14 x 10^{-4}	8.67±0.5*
4.24 µg/ml	2.4 x 10^4±11885	670±81.65	9.05±1.82	280 x 10^{-4}±41 x 10^{-4}	9.73±2.4*
**Hyoscine butylbromide**					
0.005 µg/ml	2.5 x 10^4±817	39±16.33	0.53±0.18	16 x 10^{-4}±7 x 10^{-4}	0.51±0.18*
0.01 µg/ml	2.5 x 10^4±4082	32±8.16	0.43±0.08	14 x 10^{-4}±6 x 10^{-4}	0.44±0.14*
0.02 µg/ml	2.4 x 10^4±816	28±12.25	0.38±0.2	12 x 10^{-4}±5 x 10^{-4}	0.42±0.23*
0.04 µg/ml	2.2 x 10^4±3266	19±4.08	0.26±0.08	9 x 10^{-4}±3 x 10^{-4}	0.32±0.14*
0.08 µg/ml	2.4 x 10^4±4082	15±5.72	0.20±0.06	7 x 10^{-4}±3 x 10^{-4}	0.22±0.08*
**Tiemonium methylsulfate**					
0.8 µg/ml	2.5 x 10^4±2449	74±8.16	1.00±0.19	29.6 x 10^{-4}±4 x 10^{-4}	1.01±0.12
1.6 µg/ml	2.5 x 10^4±3266	78±14.7	1.05±0.28	31 x 10^{-4}±2 x 10^{-4}	1.06±0.18
3.2 µg/ml	2.3 x 10^4±5312	83±10.6	1.12±0.23	37 x 10^{-4}±8 x 10^{-4}	1.26±0.3
6.4 µg/ml	2.5 x 10^4±4899	87±5.72	1.18±0.17	37 x 10^{-4}±5 x 10^{-4}	1.24±0.05
12.8 µg/ml	2.4 x 10^4±6532	88±13.06	1.19±0.27	40 x 10^{-4}±17 x 10^{-4}	1.42±0.74
**Levofloxacin at sub-MIC (0.125 µg/ml)**	2.2 x 10^4±1633	2500±408	33.78±8.21	1150 x 10^{-4}±253 x 10^{-4}	40.08±13.2*

Results are shown as mean ± SD, significant differences between non-antibiotic dosed samples and the control were analyzed by independent-sample *t* test.* $P < 0.05$ were considered significant.

Figure (3): Fold changes of transfer ratio under the exposure of metoclopramide HCl at different concentrations

Figure (4): Fold changes of transfer ratio under the exposure of tiemonium methyl sulfate at different concentrations

Figure (5): Fold changes of transfer ratio under the exposure of hyoscine butyl bromide at different concentrations.

3.3 Reverse conjugation experiment in the presence of non-antibiotic agents:

To test if the transconjugant is transferable, the reverse conjugative experiment is performed. The plasmid is transferred from conjugants to donners. It was seen that the conjugative transfer frequency was enhanced in the presence of metoclopramide HCl as compared to negative control. Also, an increases in the fold changes of the transfer frequency occurred in the presence of the tested drug. Transconjugant colonies were chosen and re-grown on selection plates to verify the identity of the recipient cells.

3.4 Determination of Minimum inhibitory concentration of transconjugants:

The MICs of the transconjugants against tetracycline and streptomycin were detected (Table 3) and were the same or higher than the donor or recipients MICs.

Table (3): Minimum inhibitory concentrations (MICs) of donor, recipient, and different transconjugants towards antibiotics

**Antibiotics**	**MICs (mg/L)**				
	**Donor**	**Recipient**	**TC 1**	**TC 2**	**TC 3**
**Tetracycline**	32	4	32	32	32
**Streptomycin**	4	64	64	64	64

*TC 1-3: transconjugants in mating system treated with hyoscine butylbromide, metoclopramide HCl and tiemonium methyl sulfate, respectively.

3.5 Detection of plasmids:

Confirmation of successful conjugal transfer was done by plasmid analysis on agarose gel electrophoresis. Conjugative plasmid was detected in donor and transconjugant strains as illustrated by first and second lanes (Figure 6). Recipient isolate did not show any plasmid as recorded in lane 3.

Figure (6): Plasmid detection in donor (lane 1), transconjugants (lane 2) and recipient (lane 3)

3.6 Effect of non-antibiotic pharmaceuticals on conjugation efficiency:

Figure 4 illustrates TEM images of conjugative transfer in the presence of non-antibiotic pharmaceuticals and in their absence as a control (Fig. 7A1,2). Metoclopramide HCl induced conjugal transfer between donor and recipient as seen in the close contact between cells, periplasmic bridges and aggregation of cells (Fig. 7B1,2). Hyoscine butylbromide decreased conjugal activity as observed in the little affinity between cells (Fig. 7C).

Figure (7): TEM images for conjugal activity between donor and recipient A) Control, B) In presence of metoclopramide HCl, C) In presence of hyoscine butyl bromide

4. Discussion

The present findings highlight the effect of non-antibiotic pharmaceuticals on bacterial behavior and how this will influence the dissemination of antibiotic resistance between isolates changing the duration of activity of used antibiotics. Antibiotic resistance is considered a major health problem which arose mainly due to the misuse of antimicrobial agents all over the world. This dilemma is usually attributed to various causes such as: availability of antibiotics everywhere and health care persons may suffer from lack of education. Also, many patients stop treatment with antibiotics upon feeling better, use leftover antibiotics and may use antibiotics in treating viral infectio[illegible][29-31]. The rapid dissemination of antibiotic resistance genes (ARGs) among bacterial species occurs through horizontal gene transfer (HGT). Conjugation is a common mechanism by which bacteria horizontally transfer their resistance genes for adaptation with stress conditions such as continuous antibiotic exposure and lack of nutrients **[32]**. Bacterial conjugation is a major promoter for bacterial genome evolution which supply bacteria by required genes for antibiotic resistance, biofilm formation, virulence and heavy metal resistance **[33, 34]**. Sub-inhibitory concentration of antibiotics has been found to induce dissemination of antibiotic resistance by HGT particularly through conjugation and transformation **[11, 35].** Not only antibiotics affect gene transfer between bacteria but also non-antibiotic pharmaceuticals may be supposed to have an effect as recorded for more than 200 non-antibiotics which influence gut bacteria like antibiotics do **[15]**. All over the world, non-antibiotic pharmaceuticals represent 95% of the total drugs in the market. Although their wide distribution, little is known about how non-antibiotic pharmaceuticals affect HGT between bacteria **[36, 37]**. In the present work, effect of metoclopramide HCl, hyoscine butylbromide and tiemonium methylsulfate on conjugal transfer between *E. coli* isolates was studied. Also, levofloxacin at sub-inihibitory concentration was included. Hyoscine butylbromide and tiemonium methylsulfate have antispasmodic activity while metoclopramide HCl has antiemetic effect. Metoclopramide HCl has antibiotic-like effect behavior as for promoting conjugal transfer between isolates especially when antibiotic is administered at sub-inhibitory concentration. While conjugal transfer frequency is dramatically increased by metoclopramide HCl, it was obviously suppressed by hyoscine butylbromide. This means that co-administration of

metoclopramide HCl as antiemetic with antibiotics may promote the transfer of antibiotic resistance genes among pathogenic bacteria in the GIT decreasing the susceptibility to the antibiotic in the long run use. Acceleration of dissemination of antibiotic resistance was reported for non-antibiotic pharmaceuticals such as ibuprofen, naproxen, diclofenac, gemfiprozil and propranolol as plasmid-borne conjugal transfer was promoted between bacteria of the same and different genera **[11]**. Also, dissemination of plasmid-mediated antibiotic resistance was induced by the antiepileptic carbamazepine **[38]**. These findings agree with what was obtained in our study for metoclopramide HCl. Norepinephrine has not revealed any effect on plasmid conjugal transfer between E.coli isolates **[39]**. The previous result agrees with what achieved in the present work for tiemonium methylsulfate which caused a slight increase in conjugal transfer between *E. coli* isolates. In the present study, hyoscine butylbromide dramatically decreased conjugal transfer between tested *E. coli* supporting the activity of co-administered antibiotics by weakening the resistance of targeted pathogenic bacteria. Expired drug metoclopramide HCl indicated antibacterial activity for carbon steel in 0.5 M H3PO4 solution **[40]** which disagrees with what achieved in the present work as none of the tested non-antibiotics revealed antibacterial activity against isolates. TEM imaging was applied to reveal changes of cell arrangement as an indicator to study the effect of metoclopramide HCl and hyoscine butylbromide on bacterial cells. Aggregation of cells, formation of periplasmic bridges and decreasing distances between cells were obviously noticed with metoclopramide HCl while with hyoscine butylbromide little affinity between cells was observed. In absence of non-antibiotic pharmaceuticals, cells were separated and intact but in presence of non-antibiotics, cells became closer to each other with partially damaged cell membrane **[11]**. In the present findings, the role of non-antibiotic pharmaceuticals in dissemination of antibiotic resistance between pathogenic gastrointestinal tract *E. coli* isolates was clearly demonstrated. Previous findings turn on alarm for bacterial response towards different administered pharmaceuticals highlighting the bacterial behavior when non-antibiotics are co-administered with antibiotics. More work is required to illustrate the effect of pharmaceuticals in the market on the transfer of resistance genes and how this would contribute to maximize or minimize the antibiotic resistance crisis.

5. Conclusion

In prescribing antibiotics for treating intestinal pathogenic *E.coli*, antiemetic metoclopramide HCl was found to increase the spread of resistance against the co-administered antibiotic by promoting the plasmid-mediated conjugal transfer of antibiotic resistance genes followed by tiemonium methylsulfate . While, antispasmodic hyoscine butylbromide showed lower rate of conjugal transfer in comparison to contro

Author contribution

Formal analysis, Alaa Alqahtani and Eman Farouk; Investigation, Sahar Ghanem and Eman Farouk; Methodology, Doaa Safwat Mohamed, Mohamed El-Mokhtar, Sahar Ghanem, Ramadan Yahia, Mohammed A. S. Abourehab and Eman Farouk; Project administration, Doaa Safwat Mohamed; Software, Mohammed A. S. Abourehab; Supervision, Rehab Mahmoud Abd El-Baky and Alaa Alqahtani; Validation, Doaa Safwat Mohamed and Mohamed El-Mokhtar; Visualization, Mohamed El-Mokhtar and Ramadan Yahia; Writing – original draft, Rehab Mahmoud Abd El-Baky, Sahar Ghanem, Ramadan Yahia, Alaa Alqahtani, Mohammed A. S. Abourehab and Eman Farouk.

Funding: This work was funded by the Deanship of Scientific Research at Umm Al-Qura University by Grant Code: (**23UQU4290565DSR058**).

Institutional Review Board Statement The study was conducted according to the guidelines of the Declaration of Helsinki, priori approval (No. 2/2022) by the ethical committee of Faculty of Pharmacy, Deraya University.

Informed Consent Statement

Not applicable.

Data Availability Statement

Not applicable.

Acknowledgments: The authors would like to thank the Deanship of Scientific Research at Umm Al-Qura University for supporting this work by Grant Code: (**23UQU4290565DSR058**).

Conflicts of Interest

The authors declare no conflict of interest.

References

1. Organization WH. Antimicrobial resistance: global report on surveillance: World Health Organization; 2014.
2. Levy SB, Marshall B. Antibacterial resistance worldwide: causes, challenges and responses. Nature medicine. 2004;10(12):S122-S9.
3. O'Neill J. Review on antimicrobial resistance. Antimicrobial resistance: tackling a crisis for the health and wealth of nations. 2014;2014(4).
4. Fernández L, Hancock RE. Adaptive and mutational resistance: role of porins and efflux pumps in drug resistance. Clinical microbiology reviews. 2012;25(4):661-81.
5. Johnston C, Martin B, Fichant G, Polard P, Claverys J-P. Bacterial transformation: distribution, shared mechanisms and divergent control. Nature Reviews Microbiology. 2014;12(3):181-96.
6. El-Sayed Z, Al-Ghamdi AK, Azhar EI, Khalifa NA, Ashshi AM, Faidaha HS. Multidrug resistant bacterial strains and their associated plasmid profile. Life Sci J. 2015;12(1).
7. Olaniyi TD, Adetutu A. In silico anti-quorum sensing activities of phytocompounds of Psidium guajava in Salmonella enterica serovar Typhi. Journal of Umm Al-Qura University for Applied Sciences. 2023. doi: 10.1007/s43994-023-00029-6.
8. Llosa M, Gomis-Rüth FX, Coll M, Cruz Fdl. Bacterial conjugation: a two-step mechanism for DNA transport. Molecular microbiology. 2002;45(1):1-8.
9. Maheshwari M, Abulreesh HH, Khan MS, Ahmad I, Pichtel J. Horizontal gene transfer in soil and the rhizosphere: impact on ecological fitness of bacteria. Agriculturally important microbes for sustainable agriculture: Springer; 2017. p. 111-30.
10. Von Wintersdorff CJ, Penders J, Van Niekerk JM, Mills ND, Majumder S, Van Alphen LB, et al. Dissemination of antimicrobial resistance in microbial ecosystems through horizontal gene transfer. Frontiers in microbiology. 2016;7:173.
11. Wang Y, Lu J, Zhang S, Li J, Mao L, Yuan Z, et al. Non-antibiotic pharmaceuticals promote the transmission of multidrug resistance plasmids through intra-and intergenera conjugation. The ISME journal. 2021;15(9):2493-508.
12. Baym M, Stone LK, Kishony R. Multidrug evolutionary strategies to reverse antibiotic resistance. Science. 2016;351(6268):aad3292.
13. Wang Q, Peng K, Liu Y, Xiao X, Wang Z, Li R. Characterization of TMexCD3-TOprJ3, an RND-type efflux system conferring resistance to tigecycline in Proteus mirabilis, and its associated integrative conjugative element. Antimicrobial agents and chemotherapy. 2021;65(7):e02712-20.
14. Manufacturers IFoP, Associations. The Pharmaceutical Industry and Global Health. Facts and Figures 2017. 2017.
15. Maier L, Pruteanu M, Kuhn M, Zeller G, Telzerow A, Anderson EE, et al. Extensive impact of non-antibiotic drugs on human gut bacteria. Nature. 2018;555(7698):623-8.
16. Bottery MJ, Wood AJ, Brockhurst MA. Temporal dynamics of bacteria-plasmid coevolution under antibiotic selection. The ISME journal. 2019;13(2):559-62.
17. Kaper JB, Nataro JP, Mobley HL. Pathogenic escherichia coli. Nature reviews microbiology. 2004;2(2):123-40.
18. Schuetz AN, editor Emerging agents of gastroenteritis: Aeromonas, Plesiomonas, and the diarrheagenic pathotypes of Escherichia coli. Seminars in Diagnostic Pathology; 2019: Elsevier.

19. Hassannezhad M, Hosseini M, Ganjali MR, Arvand M. Electrochemical Sensor Based on Carbon Nanotubes Decorated with ZnFe2O4 Nanoparticles Incorporated Carbon Paste Electrode for Determination of Metoclopramide and Indomethacin. ChemistrySelect. 2019;4(25):7616-26.
20. Devi OZ, BAsAvAIAH K, Vinay KB. Determination of metoclopramide hydrochloride in pharmaceuticals and spiked human urine through diazotization reaction. Journal of Food and Drug Analysis. 2012;20(2).
21. Chen G-L, Hsu W-H. Hyoscine-N-butyl-bromide-induced hypotension and myocardial ischemia. Case Reports in Critical Care. 2013;2013.
22. Islam MS, Islam MS, Rafiquzzaman M, Kundu SK. UV-Spectroscopic method for estimation of Tiemonium methylsulfate 50 mg tablet in bulk and pharmaceutical preparations. International Journal of Pharmaceutical Sciences and Research. 2014;5(2):548.
23. Sunde M, Norström M. The genetic background for streptomycin resistance in Escherichia coli influences the distribution of MICs. Journal of Antimicrobial Chemotherapy. 2005;56(1):87-90.
24. Zhang Q, Lambert G, Liao D, Kim H, Robin K, Tung C-k, et al. Acceleration of emergence of bacterial antibiotic resistance in connected microenvironments. Science. 2011;333(6050):1764-7.
25. Harley J, Prescott L. Laboratory Exercises in Microbiology 5th Edition The McGraw-Hill. Companies. 2002.
26. Shun-Mei E, Zeng JM, Yuan H, Lu Y, Cai RX, Chen C. Sub-inhibitory concentrations of fluoroquinolones increase conjugation frequency. Microbial pathogenesis. 2018;114:57-62. Epub 2017/11/28. doi: 10.1016/j.micpath.2017.11.036. PubMed PMID: 29174700.
27. Guo J, Gao S-H, Lu J, Bond PL, Verstraete W, Yuan Z. Copper oxide nanoparticles induce lysogenic bacteriophage and metal-resistance genes in Pseudomonas aeruginosa PAO1. ACS applied materials & interfaces. 2017;9(27):22298-307.
28. Benjamini Y, Hochberg Y. Controlling the false discovery rate: a practical and powerful approach to multiple testing. Journal of the Royal statistical society: series B (Methodological). 1995;57(1):289-300.
29. O Nour M, Mohamed K, AL Bishi A, Amir M. INFECTION CONTROL AMONG HEALTHCARE PROVIDERS AT UMM AL QURA UNIVERSITY MEDICAL CENTER, MAKKAH, SAUDI ARABIA. A CROSS-SECTIONAL STUDY. Al-Azhar Medical Journal. 2017;46(2):515-30.
30. Zahran SE, Abdelhafez AM, Rashidove A. Implementing the WHONET Program for Surveillance of Antimicrobial Resistance in One Hospital in Makkah, Saudi Arabia. World Applied Sciences Journal. 2018;36(8):976-88.
31. Al-Gethamy MM, Faidah HS, Adetunji HA, Haseeb A, Ashgar SS, Mohanned TK, et al. Risk factors associated with multi-drug-resistant Acinetobacter baumannii nosocomial infections at a tertiary care hospital in Makkah, Saudi Arabia - a matched case–control study. Journal of International Medical Research. 2017;45(3):1181-9. doi: 10.1177/0300060517706284.
32. Virolle C, Goldlust K, Djermoun S, Bigot S, Lesterlin C. Plasmid transfer by conjugation in gram-negative bacteria: from the cellular to the community level. Genes. 2020;11(11):1239.
33. Lawrence JG. Gene transfer, speciation, and the evolution of bacterial genomes. Current opinion in microbiology. 1999;2(5):519-23.
34. Ochman H, Lawrence JG, Groisman EA. Lateral gene transfer and the nature of bacterial innovation. nature. 2000;405(6784):299-304.
35. Wang Y, Lu J, Engelstädter J, Zhang S, Ding P, Mao L, et al. Non-antibiotic pharmaceuticals enhance the transmission of exogenous antibiotic resistance genes through bacterial transformation. The ISME Journal. 2020;14(8):2179-96.
36. Hamad B. The antibiotics market. Nature reviews Drug discovery. 2010;9(9):675.

37. Widdus R. Public–private partnerships for health: their main targets, their diversity, and their future directions. Global Health: Routledge; 2017. p. 431-8.
38. Wang Y, Lu J, Mao L, Li J, Yuan Z, Bond PL, et al. Antiepileptic drug carbamazepine promotes horizontal transfer of plasmid-borne multi-antibiotic resistance genes within and across bacterial genera. The ISME journal. 2019;13(2):509-22.
39. Alhaadi M. Effect of norepinephrine on conjugation of Escherichia coli strains. 2013.
40. Anaee RA, Abd Al-Majeed MH, Naser SA, Kathem MM, Ahmed OA. Antibacterial inhibitor as an expired metoclopramide in 0.5 M phosphoric acid. Al-Khwarizmi Engineering Journal. 2019;15(1):71-81.

Figure Click here to access/download;Figure;Figures.docx

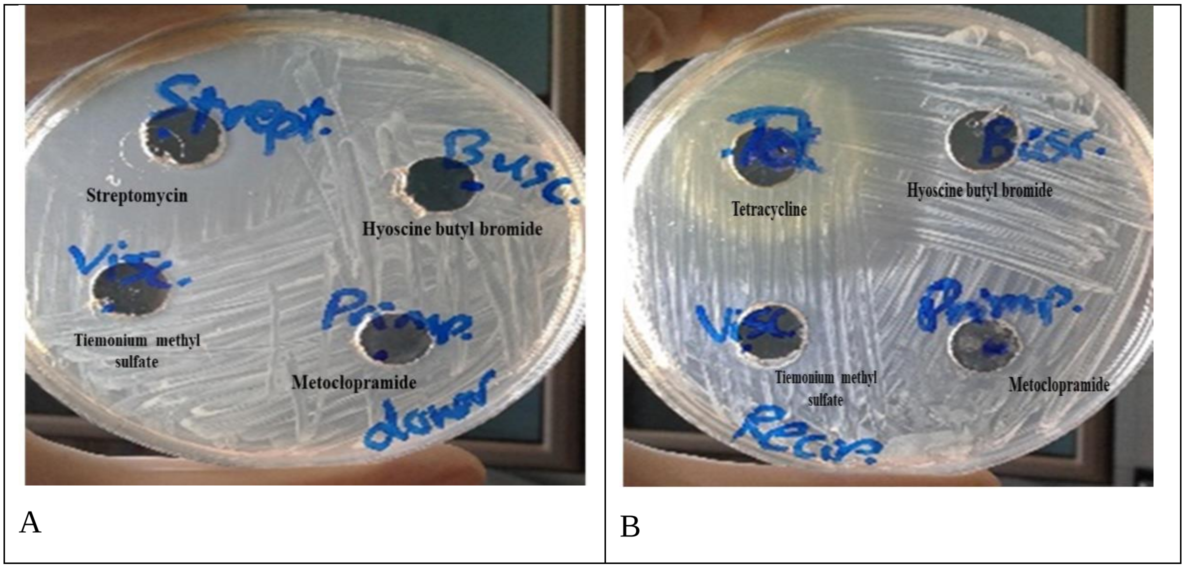

Figure 1: Antibacterial activity of non-antibiotic pharmaceuticals: A) against donor isolate, B) against recipient isolate.

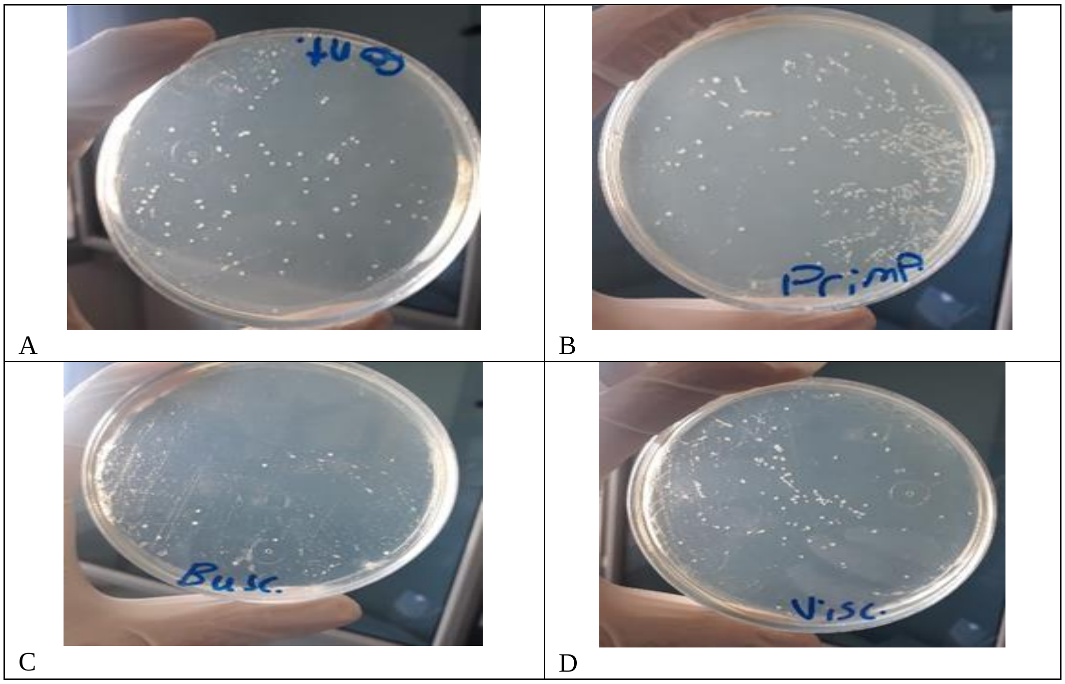

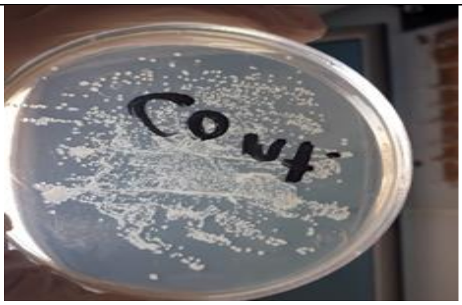

E

Figure (2): Effect of antibiotics and non-antibiotic pharmaceuticals on conjugal transfer frequency: A) Control, B) Metoclopramide HCl, C) Hyoscine butylbromide D) Tiemonium methylsulfate, E) Levofloxacin at sub-MIC

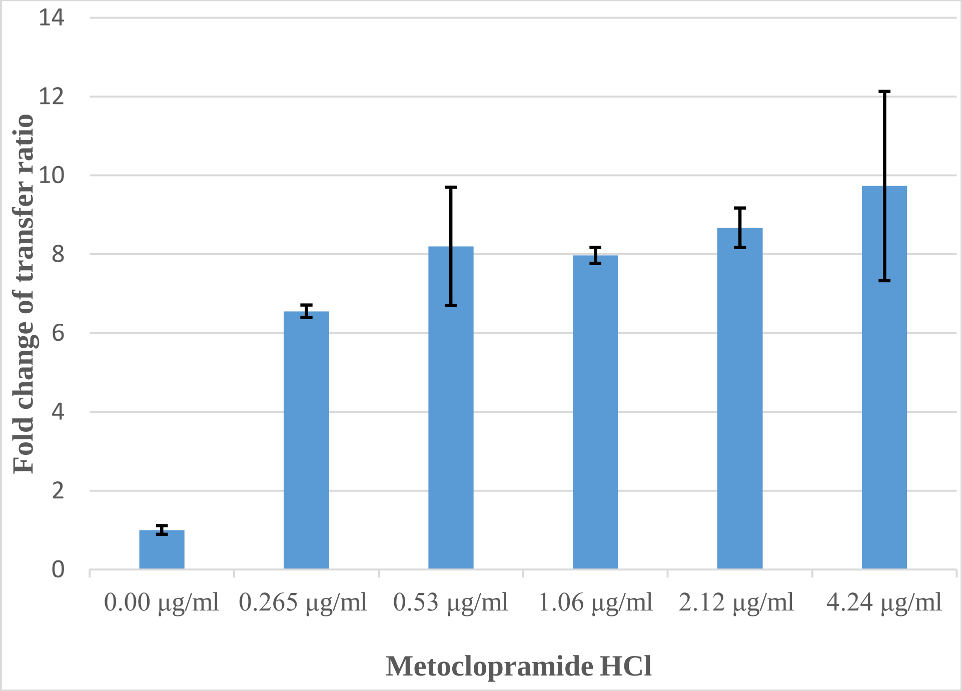

Figure (3): Fold changes of transfer ratio under the exposure of metoclopramide HCl at different concentrations

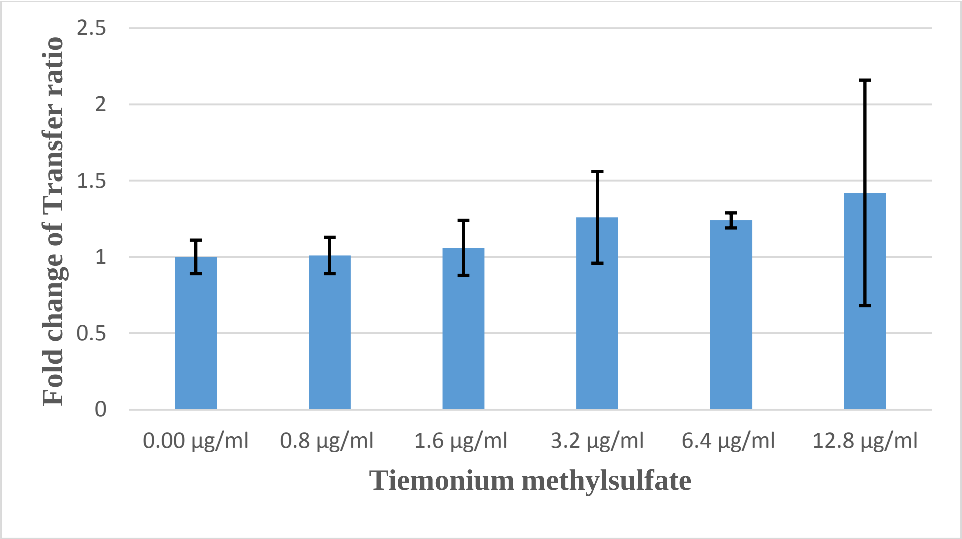

Figure (4): Fold changes of transfer ratio under the exposure of tiemonium methyl sulfate at different concentrations

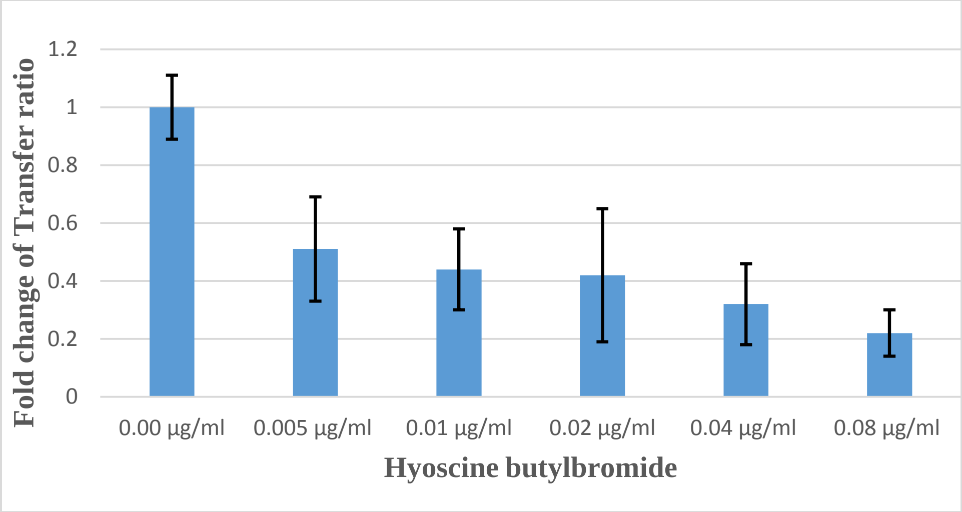

Figure (5): Fold changes of transfer ratio under the exposure of hyoscine butyl bromide at different concentrations

.

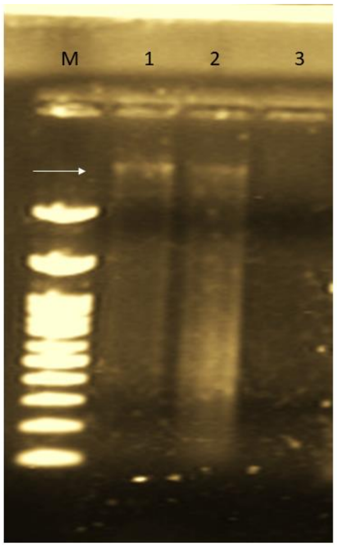

Figure (6): Plasmid detection in donor (lane 1), transconjugants (lane 2) and recipient (lane 3)

A	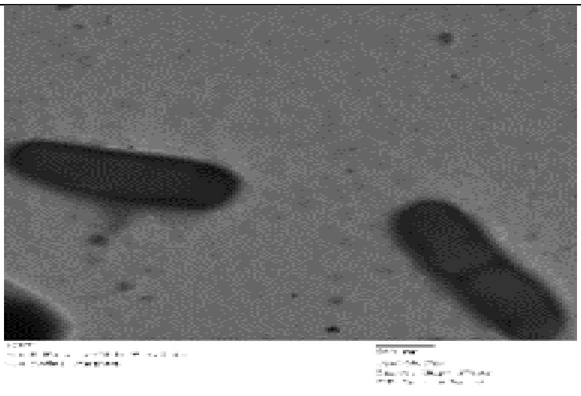	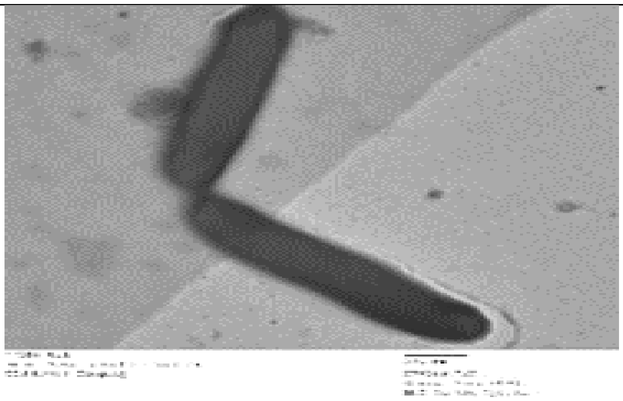
B	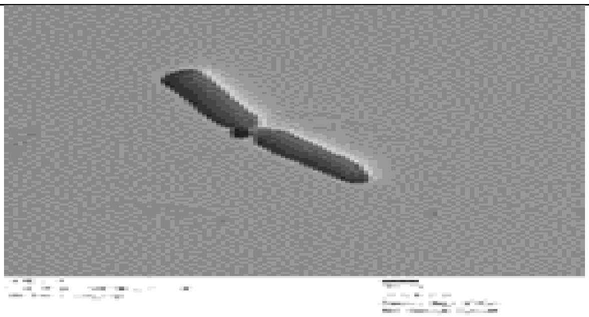	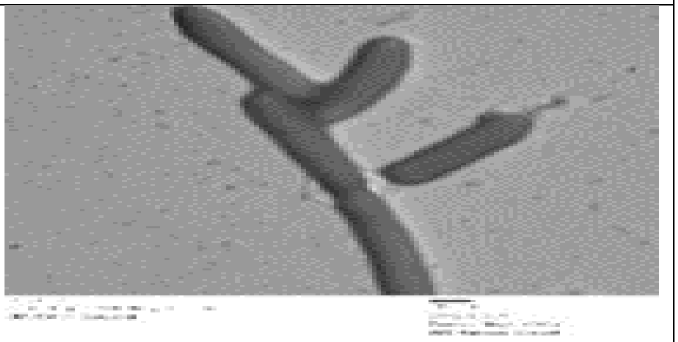

C	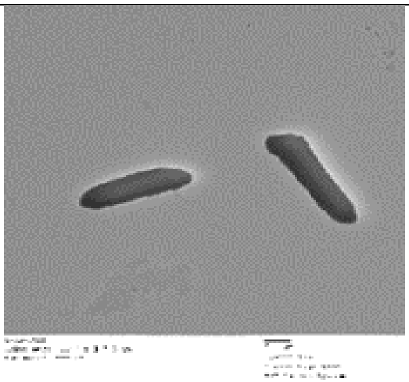

Figure (7): TEM images for conjugal activity between donor and recipient A) Control, B) In presence of metoclopramide HCl, C) In presence of hyoscine butyl bromide
