# Supplementary material for: Influence of selected non-antibiotic pharmaceuticals on antibiotic resistance gene transfer in Escherichia coli
Source: PLoS One. 2024 Jun 21;19(6):e0304980. doi: 10.1371/journal.pone.0304980 (PMC11192386; doi:10.1371/journal.pone.0304980)
Supplement: Attachment — Submitted filename: Response to reviewers (6).docx [file pone.0304980.s004.docx]

**Response to reviewers**

| **Reviewer 1 comment** | **Response** |
| --- | --- |
| Dear authors, You are doing well. Please track the requested revisions on the attached file. Your good tracking and revising all the comments to create the manuscript is so suitable for the publication | Thank you for your comment  the requested revisions on the attached file are done.  The suggested references were added. |
| **Reviewer 2 comment** | **Response** |
| Write, ‘..to be used as donor and recipient, respectively.’ | Done |
| First paragraph of discussion, write “..The rapid dissemination of ARGs among bacterial species occurs through HGT.’ | Done |
| **Reviewer 3 comment** | **Response** |
| The manuscript investigates the role of certain antiemetics and antispasmodics in disseminating antibiotic-resistance genes among pathogenic E. coli in the gastrointestinal tract. It presents an important study on the effect of non-antibiotic pharmaceuticals on the conjugal transfer of resistance genes. | Thank you very much |
| No line numbers and it wasn't easy to review. | Line numbers are added |
| Maybe authors could use the word antimicrobial instead of antibiotics to improve breadth and context. | Done |
| The manuscript could be further improved by improving English language usage. | Manuscript was revised |
| It would have been great if the authors had explained the selection criteria for the non-antibiotic pharmaceuticals studied and their relevance to antibiotic resistance. | It is explained in the Discussion section. Starting from line 312.  As antiemetic and antispasmodic drugs are widely used by patients during their course of treatment of GIT infection caused by *E. coli*. So, we thought to test their effect on the development by the transfer of resistance genes. |
| Check all abbreviations have a full form. For example, the word GIT is written only in abbreviated form. | Done |
| The meaning of the sentence:. Surprisingly, it was recently documented that the consumption of non-antibiotic prescribed drugs represents about ninety fifth of the drug market [14],…." is confusing.  Please use more technical words like detection rate, prevalent, rather than ….." are highly detected [18]." | The sentence is revised |
| The existing knowledge on the role of antiemetics and antispasmodics on antimicrobial resistance development and how the current study is going to fill the existing knowledge gap isn't clear. | A paragraph is added in the Introduction section |
| More emphasis is given to antibiotics rather than antiemetics and antispasmodics, especially in the first paragraph. | We discuss the main problem of antibiotic resistance and horizontal gene transfer through conjugation and the effect of non-antibiotics pharmaceuticals like antiemetics and antispasmodics on gene transfer. |
| Some sentences lack citation. For example: "The previous result agrees with what was achieved in the present work for tiemonium methylsulfate, which caused a slight increase in conjugal transfer between E. coli isolates." | The citation already presents at the end of previous sentence and is added again. |
| I recommend further elaborating on the mechanisms by which metoclopramide HCl enhances the conjugal transfer of resistance genes and the implications of these findings in a clinical setting. | Many researches write on the effect of non-antibiotic pharmaceutical on the dissemination and they reported that the mechanism is still unknown but it may be due to their induction of reactive oxygen species (ROS) in the donor cell which enhance the conjugal transfer of resistance genes and these drugs only may increase the membrane permeability to large plasmid.^1^,^2^ |
| **Reviewer 4 comment** | **Response** |
| Which standard protocols were used by authors for MIC? | MIC of tetracycline and streptomycin against donor, recipient and conjugants was performed by agar well diffusion method according to CLSI. |
| English editing is highly recommended by a native speaker. Some part is difficult to understand. | It was revised |
| All figure legends must be clear and informative. | Figure legends are revised |
| Table 1 is not necessary. Put it in Suppl. Files | Table 1 is attached as supplementary file |
| In figure 1: Which culture method used? | Agar well diffusion method is used to test the antibacterial activity of the tested drugs against donor and recepient |
| Figure 6: the plasmid band is vague. replace with better fig. | This is the only picture that we have taken due to the poor resolution power of the camera we have. If it is unclear wee can remove it from results. |
| Figure 7: The quality is poor. the bar should be added for TEM. | Figure 7 images were replaced by high quality image with bars |

1.	Wang Y, Yu Z, Ding P, et al. Non-antibiotic pharmaceuticals promote conjugative plasmid transfer at a community-wide level. *Microbiome.* 2022;10(1):124.

2.	Wang Y, Lu J, Zhang S, et al. Non-antibiotic pharmaceuticals promote the transmission of multidrug resistance plasmids through intra-and intergenera conjugation. *The ISME journal.* 2021;15(9):2493-2508.
