# Supplementary material for: Influence of selected non-antibiotic pharmaceuticals on antibiotic resistance gene transfer in Escherichia coli
Source: PLoS One. 2024 Jun 21;19(6):e0304980. doi: 10.1371/journal.pone.0304980 (PMC11192386; doi:10.1371/journal.pone.0304980)
Supplement: Attachment — Submitted filename: Response 2 to reviewers (1).docx [file pone.0304980.s006.docx]

**Response to reviewers**

| **Reviewer comment** | **Response** |
| --- | --- |
| **General comments** | |
| General comments: A thorough proofreading by a native English speaker or professional editor is recommended to refine the language. A few typographical errors and grammatical issues throughout the text need correction. For instance, consistency in the use of terminology such as "non-antibiotics" versus "non-antibiotic pharmaceuticals" should be addressed. The words antibiotic and antimicrobial are both used interchangeably in the manuscript. Use one term/word consistently to explain one thing. Some sentences are overly complex and could be simplified for better readability. | "non-antibiotics" versus "non-antibiotic pharmaceuticals" is addressed. One term/word is used consistently to explain one thing |
| **Title and abstract** | |
| Title: The current title could be revised for improvement. A proposed revision might be: "Influence of Selected Non-Antibiotic Pharmaceuticals on Antibiotic Resistance Gene Transfer in Escherichia coli." | Done. We changed the title to the proposed one |
| **Introduction** | |
| The introduction outlines the background and the significance of the research. It would enhance the manuscript to include a brief explanation of previous studies directly linking non-antibiotic pharmaceuticals with conjugative transfer, providing a stronger justification for the current study. | Done |
| Line 80: the words prevalent and rate are the same here. Use detection rate or prevalent. | Done |
| **Materials and methods** | |
| Line 109: what does “Raw materials” refer to? | Refer to the used antibiotics. It is deleted |
| Line 109-113: in some instances, product numbers are indicated in parenthesis, while in others, the country where the product is made is indicated. Please revise and follow the journal guidelines. | Done |
| **Results** | |
| Was there any dose-based response? If applicable, describe the dose-response relationships and whether a threshold effect is observed for the pharmaceuticals under study. | Yes and it is described in the result section |
| **Discussion** | |
| The entire discussion is written as one paragraph, making it difficult to follow the points. Please revise the discussion part by making each main point a paragraph. | Done |
| The discussion effectively ties the results back to the broader implications of antibiotic resistance. Expanding on the molecular mechanisms potentially involved in the observed effects could enrich the discussion. For example, are there known interactions at the cellular or molecular level between the pharmaceuticals studied and bacterial cell membranes or DNA transfer machinery? | Yes and the interactions at the cellular or molecular level between the pharmaceuticals and DNA transfer is discussed in the Discussion section |
